# Editorial: Prevention of musculoskeletal pain among professional drivers

**DOI:** 10.1002/1348-9585.12170

**Published:** 2020-10-06

**Authors:** Ming‐Lun Lu

**Affiliations:** ^1^ National Institute for Occupational Safety and Health (NIOSH) Cincinnati OH USA

*J Occup Health* published a systematic review of the prevalence of musculoskeletal pain among professional drivers.^1^ The meta 12‐month prevalence estimates of musculoskeletal pain for specific body regions were calculated with a total sample size of 18 882 respondents. The sample was pooled from 56 cross‐sectional, case‐control, or prospective cohort studies from 23 countries that reported the prevalence of musculoskeletal pain in professional drivers. Findings of the review show that the low back was the most frequently reported body region for musculoskeletal pain with a meta mean prevalence rate of 53% (N = 9998), followed by neck (42.4%, N = 3480), shoulder (39.2%, N = 2751), and other body regions. Collectively, the prevalence rates of musculoskeletal pain indicate that professional drivers are at an increased risk of developing musculoskeletal pain in many body regions, compared with other occupational groups.

The review was the first to calculate the meta prevalence rates of musculoskeletal pain for nine body regions in professional drivers. The findings highlighted the severity of the global musculoskeletal problem in professional drivers, in particular, low back pain (LBP). It, however, did not review the magnitudes of the musculoskeletal problem in terms of sickness absence, disability, total healthcare cost, and other societal burden. LBP is the pre‐cursor for underlying spinal or musculoskeletal disorders (MSDs), which have caused significant burden to individuals and the society as a whole. The Global Burden of Disease 2017 study demonstrated that among 354 diseases and injuries, LBP was ranked highest in terms of leading casue of disability and years lived with disability.^2^ Katz estimated the total costs of LBP in the United States exceed $100 billion per year, with approximately one third accounted for by direct medical expenses and two thirds resulting from indirect costs, such as productivity loss and sickness absence.^3^

Despite the absence of information on the direct economic impact of musculoskeletal pain in professional drivers, the high prevalence of musculoskeletal pain among professional drivers warrants research into interventions that are effective in reducing the risk factors for musculoskeletal pain. To devise effective interventions, understanding the contributing factors of musculoskeletal pain in various body regions is imperative. The studied physical risk factors for musculoskeletal pain in professional drivers are prolonged and constrained sitting, whole body vibration (WBV) from the seat, and the long duration of driving.^4^ In many studies, LBP is associated with an increasing number of driving hours per day and length of employment. Seating comfort and design is often studied for reducing the risk factors associated with vibration and poor spinal posture (ie, loss of lordosis). Work‐related psychosocial factors have been linked to LBP among professional drivers. High job strain, effort‐reward imbalance, lack of supervisory support, and organizational injustice are typical psychosocial stressors associated with an increased risk of LBP.^5^ Among professional drivers, taxi and truck drivers may be engaged in baggage and manual materials handling, respectively. These additional physical exertions increase their exposure to the physical risk factors for musculoskeletal pain. It should be noted that most of the evidence in the literature described above is based on cross‐sectional studies with a limited power of inferring causal pathways from the risk factors.

The review in this issue indicates a lack of prospective studies investigating the relationship between the incidence of musculoskeletal pain and contributing factors. Four of five prospective studies found in the literature came from Dr Bovenzi's research group. Their 2006 study investigating the dose‐response relationship between WBV and three LBP outcome measures (12‐month incidence of LBP, high pain intensity, and LBP disability) provides significant evidence in the dose‐response relationship. That recent study supports one of the main findings in a landmark review conducted by the National Institute for Occupational Safety and Health (NIOSH) in 1997. That is, after controlling personal, workplace psychosocial factors and other physical risk factors, there is strong evidence that a dose‐response relationship between WBV and LBP exists.

Please notice that the psychosocial factors in Dr Bovenzi's series of studies were not found to be significantly associated with the 1‐year incidence of various LBP outcomes. This finding about the effects of psychosocial factors on LBP is inconsistent with that from an earlier prospective study reported by Krause et al in 1997. In Krause et al's study, both physical and psychosocial factors were simultaneously and independently associated with back or neck pain. The conflicting results are most likely due to the discrepancies in the study methodologies and the definitions of muscular pain. In Krause et al's study, all physical and psychosocial variables were based on a self‐reported questionnaire, while in Bovenzi's studies, WBV was measured objectively as the main physical risk factor along with an interview‐based questionnaire survey for manual materials handling and psychosocial factors. Although both studies employed the psychosocial scales from the Karesek's job strain model, the scales and the calculation methods for the scores of the subscales were different. The sample size and follow‐up period in Bovenzi et al's 2006 study were 202 and 1‐2 years, while those in Krause et al's 1997 study were 1449 and 7.5 years, respectively. With the smaller sample size and shorter follow‐up period in Bovenzi et al's 2006 study, their study is less likely to have a sufficient statistical power to detect significant psychosocial effects. In 2004, Krause and his colleagues published another study focusing on the incidence of workers' compensation records for the first episode of LBP during 7.5 years of follow‐up period. With the more stringent definition of LBP using medically related insurance data, Krause et al found a strong association between weekly driving hours and incidence of severe low back injuries, suggesting a strong causal role of operating transit vehicles in developing low back injuries. The most interesting finding in that study is a reduction in driving hours to 20‐30 hours per week eliminated about 60% of severe low back injuries. This simple administrative control is in line with the suggested risk control strategy from a review of interventions in public transportation studies in Europe.^6^ A reduction in weekly driving hours can lead to a significant decrease in sickness absence and cost savings for the employer.^6^

Collectively, findings from these prospective studies support the case that four research areas for improving the understanding of the relationship between risk factors and MSDs in professional drivers deserve further evaluation. The first area is the utilization of emerging technologies (eg, wearable motion sensor or computer vision) to quantify the characteristics of physical risk factors, such as WBV from the seat, the driver's sitting behavior, the posture while driving, and manual materials handling tasks. The continuous monitoring of objective risk data (as opposed to questionnaire‐based risk quantifications) in professional drivers is sparse in the literature. The objective risk data may provide insightful strategies for mitigating physical risk factors effectively. Additionally, the data will help quantify accurate driving hours and behaviors for developing a precise dose‐response relationship between driving hours and MSDs. Second, the advent of autonomous vehicles in the near term may provide new ways of interventions. Research into the interactions between professional drivers and autonomous or semi‐autonomous vehicles may shed new light on interventions. Third, research is needed to investigate the roles that various aspects of psychosocial stress play in the development of MSDs. The effects of interactions between psychosocial and physical risk factors on developing MSDs in professional drivers are still unclear and may be different from those working in the manufacturing and construction industry sectors who are typically not exposed to WBV and constrained sitting. Finally, a total of 17 studies were rated high quality (ie, low risk of bias) in the systematic review. However, only two high‐quality studies came from Asia, specifically, China. Unfortunately, these two high‐quality studies were of cross‐sectional design, which cannot provide causal relationships between risk factors and MSDs. To assess the global impact of MSDs in professional drivers, there is a need for MSD studies of longitudinal design that are representative of all geographic industrialized areas.

## DISCLAIMER

The author discloses no conflict of interest. The findings and conclusions in this article are those of the author and do not necessarily represent the official position of the National Institute for Occupational Safety and Health, Centers for Disease Control and Prevention.
